# Holoprosencephalie alobaire dans un contexte de syndrome polymalformatif: apport de l'imagerie, à propos d'un cas

**DOI:** 10.11604/pamj.2013.15.83.2797

**Published:** 2013-06-30

**Authors:** Dia Aliou Amadou, D'Almeida Franck, Mbodji Mamadou, Ka Mamadou Mourtalla

**Affiliations:** 1 Service de radiologie de l'hôpital Saint-Jean de Dieu/UFR des Sciences de la Santé, Université de Thiès, Sénégal; 2 Service de pédiatrie de l'hôpital Saint-Jean de Dieu/UFR des Sciences de la Santé, Université de Thiès, Sénégal; 3 Département de biophysique de l'UFR des Sciences de la Santé, Université de Thiès, Sénégal; 4 Département de médecine interne de l'UFR des Sciences de la Santé, Université de Thiès, Sénégal

**Keywords:** Syndrome polymalformatif, Holoprosencéphalie, Fente labio-palatine, Echographie trans-fontanellaire, tomodensitométrie, polymalformative syndrome, holoprosencephaly, Cleft lip and palate, Transfontanellar ultrasound, CT

## Abstract

L'holoprosencéphalie est une malformation cérébrale rare, d’étiologies multiples et souvent associée à des anomalies faciales évocatrices. Cette pathologie, résultant d'un défaut de développement précoce du prosencéphale, est de pronostic fœtal extrêmement réservé en particulier pour la forme alobaire. Nous rapportons à travers ce cas clinique, une holoproséncéphalie alobaire diagnostiquée à l'imagerie (ETF, tomodensitométrie) et relevée par un syndrome polymalformatif chez un nouveau-né de 03 mois.

## Introduction

L'holoprosencéphalie (HPE) correspond à une malformation congénitale grave et complexe du cerveau associée à des anomalies faciales évocatrices et particulières (hypotélorisme, cyclopie, éthmocéphalie, fente labio-palatine, etc.) [1, 2]. Elle résulte d'une anomalie du clivage du prosencéphale en hémisphères cérébraux, survenue au cours du deuxième mois gestationnel. L'HPE reste une pathologie rare, révélée parfois devant une fente labio-palatine [1, 2]. Elle est due à un défaut de clivage médian, total ou partiel, du prosencéphale à un stade embryonnaire très précoce. Les fentes labio-palatines médianes représentent l'une des malformations faciales les plus fréquentes entrant dans le cadre des holoprosencéphalies (HPE) [2].

## Patient et observation

**Figure 1:**
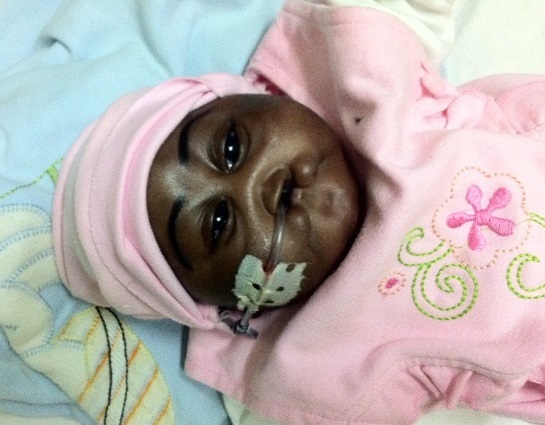
Malformation faciale complexe associant hypotélorisme et fente labio-palatine

Il s'agissait d'un nourrisson de 03 mois de sexe féminin, troisième enfant de la fratrie, suivi depuis sa naissance pour une infection néonatale sur un syndrome polymalformatif à type fente labio-palatine, d'arthrogrippose de la main droite, de microcéphalie et un hypotélorisme (Figure 1). Son statut vaccinal était à jour.

On note une grossesse mal suivie (réalisation tardive de la 1ère échographie obstétricale) et une notion de consanguinité de 2^ème^ degré. A l'examen on notait un tableau de retard staturo-pondéral sur syndrome malformatif avec une évolution satisfaisante des paramètres de croissance. Le poids à la naissance était de 2200 grammes et de 4000 grammes à 03 mois de vie. La taille était de 48 centimètres à la naissance et de 54 centimètres à 03mois. Le périmètre crânien était de 29 centimètres à la naissance et de 33 cm à 03 mois.

L’échographie cardiaque était normale. L’échographie abdominale n'objectivait pas de malformation abdominale. L’échographie trans-fontanellaire objectivait une importante hydrocéphalie avec une impression de cavité ventriculaire unique et une agénésie du corps calleux (Figure 1).

Le scanner cérébral réalisé sans injection confirmait la malformation encéphalique en mettant en évidence la microcéphalie et une holoprosencéphalie alobaire qui se manifestait par une dilatation d'une cavité ventriculaire unique associée à une fusion des thalami et une absence de la scissure inter-hémisphérique. Le parenchyme cérébral était laminé. Il s'y associait une agénésie du corps calleux et une dilatation des espaces liquidiens péri-cérébraux en rapport avec un hygroma modéré (Figure 1). L'indication d'une intervention chirurgicale de la fente labio-palatine fut posée par les chirurgiens maxillo-faciaux, mais à distance de son infection. L’évolution fut marquée par un décès du nourrisson à 4 mois de vie, dans un tableau de détresse respiratoire.

**Figure 2:**
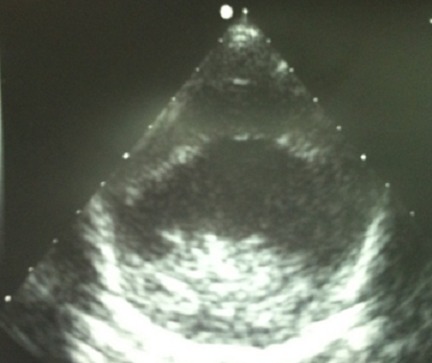
Echographie trans-fontanellaire en coupes sagittale et coronale. Dilatation du système ventriculaire, fusion des thalami et agénésie du corps calleux

**Figure 3:**
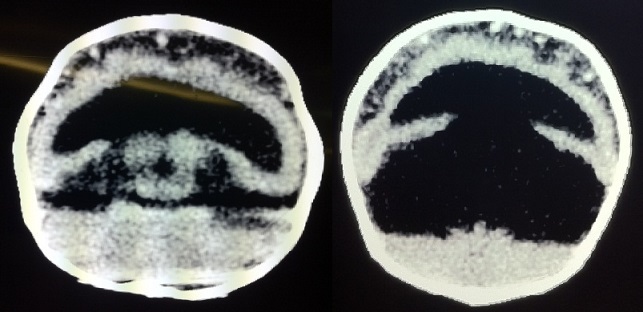
Scanner cérébral en coupes axiales. Dilatation d'une cavité ventriculaire unique avec absence de scissure inter-hémisphérique, fusion des thalami. Dilatation des espaces liquidiens péri-cérébraux en rapport avec un hygroma

## Discussion

L'holoprosencéphalie (HPE) correspond à une malformation congénitale grave et complexe du cerveau associée à des anomalies faciales évocatrices et particulières [1, 2].

La prévalence de l'HPE se situe à moins de 1 pour 10 000 naissances vivantes et une prévalence totale d'environ 1,2 pour 10 000 naissances [1, 2]. Son incidence est sous-estimée du fait du caractère abortif de la malformation ainsi que des formes mineures qui peuvent passer inaperçues. Plusieurs auteurs s'accordent sur l'association entre l’âge maternel supérieur à 30 ans et la survenue de l'HPE [3, 4], et l'absence de relation de cause à effet entre la gestité-parité et la survenue de l'HPE.

La notion de consanguinité a été rapportée dans la littérature, ainsi que l'incrimination de plusieurs mutations génétiques [3, 4]. Chez notre patiente, une notion de consanguinité de 2^ème^ degré était retrouvée. Une prédominance féminine est constatée par la majorité des auteurs [1].

L′holoprosencéphalie résulte d′un défaut d′induction du neurectoderme par la plaque préchordale, au cours de la troisième semaine de la vie embryonnaire, qui a pour conséquence une anomalie du développement du prosencéphale consistant en une absence d′évagination des vésicules prosencéphaliques responsable:

- de la présence d′une masse hémisphérique médiane remplaçant les deux hémisphères cérébraux;
- d′une absence de structures médianes, notamment de commissures;
- d′une absence de différenciation ou d′une différenciation anormale des structures dérivées des vésicules prosencéphaliques et de la vésicule diencéphalique

En fonction du degré d'individualisation des hémisphères cérébraux, trois formes anatomiques de l'HPE ont été décrites par Demyer et Zeman: alobaire, semi-lobaire et lobaire [2]. Les deux premières formes sont de très mauvais pronostic justifiant ainsi une interruption médicale précoce de la grossesse [2, 4, 5].

L'HPE alobaire est la forme la plus sévère, dans laquelle le télencéphale est constitué par une holosphère contenant une cavité ventriculaire unique fermée dans sa partie postérieure par une paroi fine qui lui confère un aspect pseudo-kystique. Les lobes olfactifs sont absents. Les thalami, petits et rudimentaires, sont fusionnés sur la ligne médiane. Une microcéphalie est présente de façon constante.

L′HPE semi-lobaire est caractérisée par l′apparition d′un sillon médian de longueur variable, marquant une séparation à la partie postérieure de l′holosphère avec ébauche de deux lobes occipitaux. Les lobes olfactifs sont absents et la microcéphalie est habituellement présente. L′HPE lobaire est la forme mineure, présentant un sillon médian qui, extérieurement, paraît séparer deux hémisphères, montrant des lobes frontaux, temporaux et occipitaux individualisés. Il ne s′agit cependant pas d′une scissure inter-hémisphérique. Sur une coupe coronale à l'imagerie (IRM ou TDM), le cortex se poursuit au fond du sillon, et même si, de chaque côté, sont ébauchées des cornes temporales et occipitales, il existe toujours une cavité ventriculaire unique dépourvue de structure médiane. Quelques fibres transversales qui semblent amorcer une commissure sont fréquemment observées au niveau de la base, mais il n′existe pas de corps calleux individualisé. Des lobes olfactifs hypoplasiques sont parfois présents.

Lorsque l′on examine l′ensemble des structures cérébrales, il apparaît que différents degrés de différenciation des noyaux gris centraux, de l′hypothalamus et des thalami peuvent être observés, sans corrélation constante avec chacun de ces trois types anatomiques [2]. D′autres types de malformation du SNC peuvent s′y associer telles que l'atrésie de l′aqueduc de Sylvius, l'hypoplasie cérébelleuse, l'agénésie vermienne, le syndrome de Dandy-Walker et les défauts de fermeture du tube neural: encéphalocèle, spina bifida avec myéloméningocèle [2].

Des anomalies oculaires sont présentes dans un quart à un tiers des cas: le développement des ébauches oculaires débute en effet, à la quatrième semaine, au niveau du prosencéphale, dans la région du futur diencéphale. Outre l’œil unique et la synophtalmie, peuvent être observés une microphtalmie, une cataracte, une dysplasie rétinienne ou un colobome irien ou rétinien [2].

L′association constante d′anomalies faciales témoigne des relations embryologiques étroites existant entre le neurectoderme et les ébauches de la face. Il existe différents phénotypes réalisant un large spectre de gravité. Dans une majorité de cas, la sévérité des anomalies faciales reflète celle des malformations cérébrales. Demyer et Zeman [2] pensent que «la face prédit le cerveau» dans environ 80% des cas. Les formes sévères telles que la cyclopie et l’éthmocéphalie correspondent habituellement à une HPE alobaire, parfois semi-lobaire. Les formes moins sévères sont caractérisées par des traits dysmorphiques [2, 6].

Les fentes labio-palatines medianes représentent l'une des malformations faciales les plus fréquentes entrant dans le cadre des holoprosencéphalies (HPE) et imposent un bilan neuro-morphologique et malformatif complet [2, 6].

Le diagnostic anténatal de l'HPE est établi grâce à l’échographie fætale [5]. Il est basé sur l'association des signes intracrâniens et d'anomalies faciales, surtout dans sa forme complète, mais cette association reste non obligatoire. Chez notre patiente, le diagnostic anténatal de la malformation a été tardif (25 semaines d'aménorrhée) du fait d'une grossesse mal suivie, ce qui contre-indiquait toute interruption thérapeutique de grossesse. Même si l'holoprosencéphalie était diagnostiquée précocement, au Sénégal l'interruption thérapeutique de grossesse reste confrontée à des problèmes d'ordre éthique, morale, juridiques et religieux.

Le diagnostic d'holoprosencéphalie alobaire chez notre patiente a été confirmé en anténatal par l'imagerie (échographie trans-fontanellaire et tomodensitométrie) en mettant en évidence une microcéphalie avec une importante dilatation d'une cavité ventriculaire unique associée à une fusion des thalami, une absence de la scissure inter-hémisphérique et une agénésie du corps calleux [1, 3].

Le pronostic était extrêmement mauvais, marqué par le décès de l'enfant à 4 mois de vie. Devant une première naissance d'un enfant atteint d'HPE, le conseil génétique s'avère intéressant afin d’évaluer les vrais risque de récurrences et d'en prévenir la survenue. Le conseil génétique chez un couple qui a déjà un enfant porteur d'une HPE doit fournir l'information précise quant à l'intérêt de la surveillance des grossesses ultérieures. Actuellement, la biologie moléculaire a fait un progrès dans l'identification de la composante génétique de cette malformation cérébrale. L’échographie de dépistage reste toujours un moyen fiable pour la surveillance de la grossesse. Dans les formes viables la chirurgie reste le seule recours. Il s'agit d'une chirurgie complexe à temps multiples [5].

## Conclusion

L'holoprosencéphalie est une pathologie f'tale rare avec une grande hétérogénéité étiologique qui peut être révélée à la naissance par une fente labio-palatine dans le cadre d'un syndrome polymalformatif. Elle résulte d'une anomalie du clivage du prosencéphale en hémisphères cérébraux, survenue au cours du deuxième mois gestationnel. Le diagnostic anténatal de l'HPE est établi grâce à l’échographie fœtale. En anténatal, l'imagerie (ETF, TDM et IRM) permet de faire un bilan lésionnel exhaustif de cette pathologie au pronostic extrêmement réservé.
